# *Roa rumsfeldi*, a new butterflyfish (Teleostei, Chaetodontidae) from mesophotic coral ecosystems of the Philippines

**DOI:** 10.3897/zookeys.709.20404

**Published:** 2017-10-18

**Authors:** Luiz A. Rocha, Hudson T. Pinheiro, Matt Wandell, Claudia R. Rocha, Bart Shepherd

**Affiliations:** 1 California Academy of Sciences, San Francisco, CA 94118, USA; 2 Current address: Monterey Bay Aquarium, Monterey, CA 93940, USA

**Keywords:** Coral triangle, deep reefs, new species, rebreather diving, reef fish

## Abstract

A new species of the butterflyfish genus *Roa* is herein described from the Verde Island Passage in the Philippines, West Pacific Ocean. *Roa rumsfeldi* **sp. n.** was found on mesophotic coral ecosystems at Puerto Galera and Batangas, and sampled through technical mixed-gas rebreather diving at 100–130 m depth. This represents the fifth known species of *Roa*. The main differences between *Roa rumsfeldi* **sp. n.** and its congeners are the lower number of pored scales in the lateral line, longer snout, longer caudal peduncle, shorter caudal fin, pelvic fin color (dark first spine vs. white in all other *Roa*), and genetics (8.4% divergence from its closest relative *Roa modesta* in the mitochondrial COI gene). *Roa* spp. are usually seen in pairs, but the two specimens we collected were solitary individuals. We have kept one of the specimens alive in the California Academy of Sciences’ Twilight Zone exhibit for more than one year, where it thrives and is feeding on a variety of dried and fresh food.

## Introduction

While collecting live specimens in Anilao, Philippines to display at the California Academy of Sciences’ Twilight Zone: Deep Reefs Revealed exhibit in 2016, LAR collected a butterflyfish identified at the time as Roa cf. modesta. After the specimen arrived in San Francisco and entered quarantine, MW noticed that the recent arrival differed from our previously collected specimens by having a black spine in the ventral fin (white in others). We went through our previous years’ collections and found a second specimen of this species collected by spear in 2015 in Puerto Galera, Philippines.

These specimens represent an undescribed species of the genus *Roa* Jordan, 1923, which currently contains four species, all from mesophotic coral ecosystems (MCEs; 30-150m depth) in the Indo-Pacific. Although some taxonomists consider this genus as a “*modestus* species complex” within *Chaetodon* (Kuiter 2004, Eschmeyer et al. 2017), *Roa* species are remarkably different from all other butterflyfishes (Tea 2015). They display high dorsal spines (usually increasing in height from spines one to three or four, and decreasing to the last), a very conspicuous color pattern (bands of brown and white), and typically occur at MCEs (Kuiter 2004).

The currently recognized species of *Roa* have largely allopatric distributions: *Roa modesta* (Temminck & Schlegel, 1844) was described from Japan and is distributed along the northwestern Pacific, recorded in the Philippines, Hong Kong, Taiwan, Ryukyu and Ogasawara Islands, and Nagasaki, in Japan (Eschmeyer et al. 2017, Froese and Pauly 2017). *Roa jayakari* (Norman, 1939) occurs in the northwestern Indian Ocean, from India to the Red Sea, while *Roa excelsa* (Jordan, 1921) is only known from the Pacific oceanic islands of Hawaii, Johnston Atoll, Pohnpei, and Guam (Eschmeyer et al. 2017). The most recently described species, *Roa australis* Kuiter, 2004, is only known from northwestern Australia, usually between 100 and 300 m depth (Kuiter 2004). Here we describe a fifth species of *Roa*, so far only found on MCEs of the Verde Island Passage in the Philippines.

## Methods

The specimens were collected using hand nets or a Hawaiian sling while diving on mixed-gas, closed-circuit rebreather (Hollis Prism 2). Counts were performed using a microscope, and morphological characters were measured to the nearest 0.1 mm following Pyle and Kosaki (2016). The last two soft rays articulating on the last complex pterygiophore in the dorsal and anal fins were counted as one ray. Morphometric and meristic data for the holotype and other *Roa* species are presented in Table 1. The specimen was deposited in the fish collection of the Philippines National Museum of Natural History (PNM), and comparative material was obtained from the California Academy of Sciences (CAS) fish collection. Mitochondrial Cytochrome c oxidase subunit I (COI) DNA was sequenced and analyzed for the new species. DNA extraction and PCR amplification of the COI were performed following Weigt et al. (2012) protocols. DNA sequences were then compared to all other *Roa* species available in GenBank (*Roa jayakari*: KF268176, KF268177, KF268178, KF268184, KF268185, KF268186; *R. modesta*: KP267584, KU944197, KU944203, KU944230). GenBank accession number for the new species is MF995631.

**Table 1.** Proportional measurements of *Roa* species. Values that do not overlap between *Roa rumsfeldi* sp.n. and the other species are in bold.

		*Roa rumsfeldi* sp. n.	*Roa australis*	*Roa excelsa*	*Roa modesta*	*Roa jayakari*
		PNM 15198	Kuiter 2004	Kuiter 2004	Kuiter 2004	CAS 73228
	Standard length	64.2 mm	69.5–119 mm	94–105 mm	51.5–97 mm	99–104.3 mm
		%	%	%	%	%
Body	depth in SL	69.8	63.6–75.0	61.0–67.0	66.5–73.2	72.5–77.5
	width in SL	15.4	14.0–19.4	14.9–17.3	15.5–19.3	15.6–16.5
Head	length in SL	41.1	36.1–42.0	34.9–37.1	31-5–40.9	40.2–40.9
Snout	length in HL	**35.9**	28.2–34.6	29.3–34.5	32.5–32.7	33.5–35.5
Eye	diameter in HL	32.2	30.1–36.1	33.6–32.5	30.8–32.6	23.6–27.1
Interorbital	width in HL	27.5	19.6–27.3	21-7–25.3	22.7–27.8	22.7–23.9
Caudal peduncle	depth in SL	10.7	11.3–14.1	10.2–11.1	12.0–13.3	10.8–11.2
	length in SL	**8.46**	4.6–6.4	4.2–4.9	4.6–5.5	6–8.2
Caudal fin	length in SL	**15.9**	21-8–25.9	20.0–21.2	21.1–25.6	19.8–22.3
Pectoral fin	length in SL	29.1	29.4–36.2	28.8–33.6	26.3–30.2	27.6–28.3
Dorsal-fin base length	spinous in SL	40.0	35.8–42.0	36.4–40.1	36.2–38.8	38.8–42.6
Dorsal-fin base length	soft in SL	31.1	31.7–42.0	31.1–33.9	35.3–40.8	33.4–33.6
Dorsal-fin spine length	1st in SL	9.1	6.2–8.8	7.0–7.7	6.4–9.3	8.4–9
	2nd in SL	18.3	13.4–21.0	15.0–17.3	15.9–22.3	16.6–18.3
	3rd in SL	24.5	35.4–21.3	33.9–36.5	23.4–30.6	24.9–25
	4th in SL	29.1	37.1–25.6	26.5–32.6	26.3–33.7	26–28.8
Dorsal-fin soft-ray length	1st in SL	**12.0**	17.6–23.9	16.6–21.2	19.5–23.4	23.2–26.3
Anal-fin base	length in SL	34.2	31.2–37.4	30.6–32.6	36.0–41.8	35.1–36.1
Anal-fin spine length	1st in SL	12.9	9.3–13.7	12.1–13.2	9.7–11.9	13.4–15.3
	2nd in SL	25.2	21.8–28.1	27.1–30.0	18.7–19.6	21.3–24.3
	3th in SL	12.8	18.9–23.0	17.2–20.8	19.6–21.9	16.7–20.4
Anal-fin soft-ray	longest in SL	13.8	17.5–23.2	18.5–22.3	17.5–26.2	21.4–23
Ventral fin	length in SL	27.5	27.5–38.1	29.9–33.0	30.2–37.8	26.2–30.8
	spine length in SL	25.2	20.3–30.2	23.6–26.0	22.3–25.8	23.3–27.7

## Results

### Roa rumsfeldi sp. n.

Taxon classificationAnimaliaPerciformesChaetodontidae

http://zoobank.org/DA519076-63AA-4866-B4D3-300C1B2F3CC8

**Figure 1.**
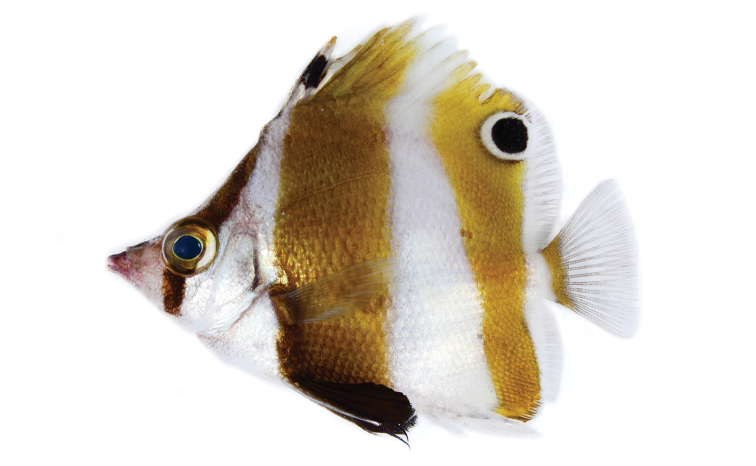
*Roa rumsfeldi* sp. n., holotype shortly after death, 77.53 mm SL, PNM 15198 (photo LA Rocha).

Figure 1 , 2 , Table 1 Deep-blackfin butterflyfish

#### Type locality.

Puerto Galera, Philippines.

#### Holotype.

PNM 15198 (Field number: HTP 506). 77.53 mm SL, GenBank accession number MF995631, Puerto Galera, Oriental Mindoro, Philippines. 13°31'17.68"N, 120°59'41.78"E, depth 110 m, collected by LA Rocha using a Hawaiian sling, 10 April 2015 (Figure 1).

#### Comparative material.

*Roa modesta* CAS 238385, CAS 27415, CAS 15627, CAS 15897; *R. jayakari* CAS 73228 (as *Chaetodon jayakari*). Data from *R. australis*, *R. excelsa* and *R. modesta* also from Kuiter (2004).

#### Diagnosis.

*Roa rumsfeldi* sp. n. differs from all of its congeners by the smaller number of pored scales in the lateral line pored scales (27 versus 37-46 in other *Roa*), longer snout (35.9% in HL vs. 28.2–35.5%), shorter first dorsal ray (12% in SL vs. 16.6–26.6%), longer caudal peduncle (8.5% in SL vs. 4.2–8.2%), shorter caudal fin (15.9% in SL vs. 19.8–25.9%), and a dark brown pelvic-fin spine (white in all other known *Roa* species).

#### Description.

Dorsal fin rays XI, 20, last soft ray branched to the base and counted as one; dorsal-fin base length: spines 40.0% in SL, and soft rays 31.1% in SL; spines increase in height from the first to the fourth: length of first spine 9.1% in SL, length of second spine 18.3% in SL, length of third spine 24.5% in SL, length of fourth spine 29.1% in SL. Soft dorsal fin follows sharp descent of rear portion of body. First soft ray 12.0% in SL. Anal-fin rays III, 17, last soft ray branched to the base and counted as one; anal-fin base 34.2% in SL; second spine very long, 25.3% in SL. Pectoral fin 13; length 29.1% in SL (Table 1). Pored lateral-line scales 27, plus 13 scales (without pores) to the peduncle. Body deep, 69.8% in SL, and compressed, 15.5% in SL; large head, length 41.1% in SL; snout long, length 35.9% in HL; eye diameter 32.2% in HL; interorbital 27.5% in HL; caudal peduncle depth 10.7% in SL, and length 8.5% in SL (Table 1).

Origin of dorsal fin above the origin of pectoral fin, posterior end of head; fin base long and mostly horizontal; soft part of dorsal fin curves downward from the third ray to caudal peduncle; soft rays posterior margin vertical; origin of anal fin below 9th spine of dorsal fin; pelvic fin with strong spine and filamentous first soft ray.

Body and head with ctenoid scales, becoming smaller towards nape and snout. Scales extending to about one third of the median fins. Tubed scales of lateral line rising at a steep angle from origin with 22 scales in an almost straight line, bending abruptly downward, ending behind posterior third of soft dorsal fin.

#### Color in life.

**Figure 2.**
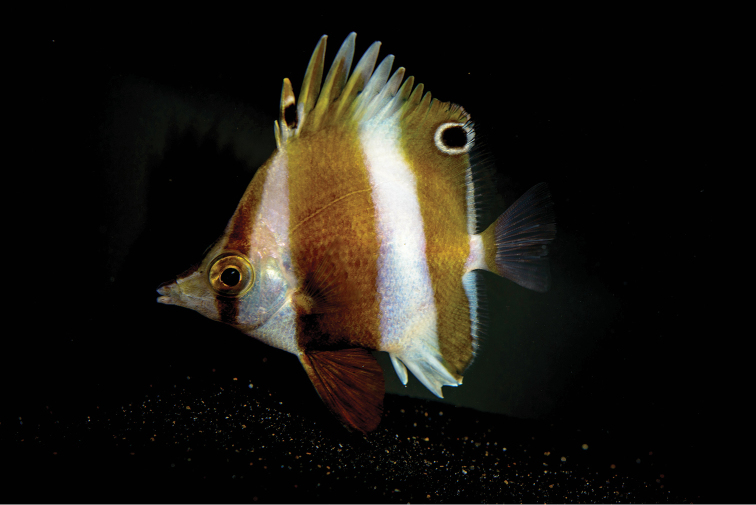
Live specimen of *Roa rumsfeldi* sp. n. photographed at the California Academy of Sciences’ Twilight Zone exhibit (photo LA Rocha).

*Roa rumsfeldi* sp. n. (Figure 1, 2) is white with three vertical dark brown bands. The first band is the narrower and runs from the origin of the dorsal fin through eye and over cheek; the second partially covers 2nd to 7th dorsal spines, going down towards the abdomen; and 3rd from the last 3–4 dorsal spines towards caudal peduncle, narrowing and covering the end of anal fin rays. Two black spots with white borders, one on the 2nd dorsal fin spine and the other on the soft dorsal fin between 2nd and 7–8th rays; all anal fin spines are white; pelvic fin, including spine, dark brown; caudal fin brown basally with translucent rays.

#### Color in alcohol.

As described above in color in life, but with lighter brown bands.

#### Etymology.

We name *Roa rumsfeldi* to honor Donald Rumsfeld who immortalized the quote: “there are known knowns; there are things we know we know. We also know there are known unknowns; that is to say we know there are some things we do not know. But there are also unknown unknowns – the ones we don’t know we don’t know.” He said that when referring to the uncertainties of war, but we think it applies perfectly to the taxonomy of MCE species: We only realized this species was new after we took a good look at it here at the aquarium in San Francisco, so we think it’s a perfect example of an unknown unknown.

#### Distribution and habitat.

*Roa rumsfeldi* sp. n. is only known to occur in the Verde Island Passage, central Philippines. It was found on MCEs of Puerto Galera, Oriental Mindoro, and Bauan, Batangas, between 100 and 130 m depth. However, the species likely has a wider distribution and remains undetected because of its preferred depth range. The ecosystems where it was found vary from sheltered rocky outcroppings heavily covered by fine sediment to areas exposed to strong currents. The ambient seawater temperature varied between 19 and 21°C during our dives, which were conducted in April-May over several years. Azooxanthellate gorgonians, black corals, and solitary stony corals are the most abundant benthic invertebrates in this habitat.

#### Comparative remarks.

In addition to the singular characteristics presented in the diagnosis section, *Roa rumsfeldi* sp. n. differs from its congeners by a lower number of dorsal soft rays (20) than *R. jayakari* (22–24); longer head (41.0% in SL) than *R. excelsa* (34.9–37.1); fewer dorsal-fin rays (20) and shorter dorsal-fin rays base (31.1% in SL) than *R. modesta* (22 rays and 35.3–40.8% in SL); and shorter 3rd anal-fin spine (12.8% in SL) and longest anal-fin ray (13.8% in SL) than *R. australis* (17.6–23.9, 18.9–23.0 and 17.5–23.2% in SL, respectively). Moreover, the COI gene sequence of *Roa rumsfeldi* does not match any other *Roa* species available at Genbank. The uncorrected genetic divergence at the COI gene between *R. rumsfeldi* and the two other available *Roa* (*R. jayakari* and *R. modesta*) is 10.5% and 8.4%, respectively.

## Discussion

Despite a few records of *Roa modesta* and *R. excelsa* in shallow waters of Japan and Hawaii, *Roa* species are normally found at depths exceeding 100 m, with trawling being the most common sampling method for these species (Kuiter 2004). As trawling is limited to bottoms lacking structural complexity, information about these reef fishes is scarce. Technical SCUBA and rebreather diving using mixed gases to explore lower MCEs (60 to 150 m) is enabling new records of reef fishes around the globe (Kane et al. 2014, Wagner et al. 2014, Pinheiro et al. 2015, Simon et al. 2016), as well as the discovery of several new species (Anderson et al. 2016, Pyle and Kosaki 2016, Pyle et al. 2016). The expansion of scientific deep diving is slowly filling our knowledge gap of the diversity and distribution of most MCE taxa, including *Roa* species. Here we consider *Roa* as a valid genus due to its morphological and niche uniqueness, however, a more detailed phylogenetic study is necessary to understand the evolutionary history of this group and its relationship with the *Chaetodon* lineage.

## Supplementary Material

XML Treatment for Roa rumsfeldi
